# New simulation model for bone formation markers in osteoporosis patients treated with once-weekly teriparatide

**DOI:** 10.1038/boneres.2014.43

**Published:** 2014-12-23

**Authors:** Sakae Tanaka, Taiji Adachi, Tatsuhiko Kuroda, Toshitaka Nakamura, Masataka Shiraki, Toshitsugu Sugimoto, Yasuhiro Takeuchi, Mitsuru Saito, John P Bilezikian

**Affiliations:** 1 Department of Orthopaedic Surgery, Faculty of Medicine, University of Tokyo, Tokyo, Japan; 2 Department of Biomechanics, Institute for Frontier Medical Sciences, Kyoto University, Kyoto, Japan; 3 Medical Affairs Department, Asahi Kasei Pharma Corporation, Tokyo, Japan; 4 National Center for Global Health and Medicine, Tokyo, Japan; 5 Research Institute and Practice for Involutional Diseases, Nagano, Japan; 6 Internal Medicine 1, Shimane University Faculty of Medicine, Izumo, Japan; 7 Toranomon Hospital Endocrine Center, Tokyo, Japan; 8 Department of Orthopaedic Surgery, Jikei University School of Medicine, Tokyo, Japan; 9 Metabolic Bone Diseases Program, Division of Endocrinology, Department of Medicine, College of Physicians and Surgeons, Columbia University, New York, USA

## Abstract

Daily 20-μg and once-weekly 56.5-μg teriparatide (parathyroid hormone 1–34) treatment regimens increase bone mineral density (BMD) and prevent fractures, but changes in bone turnover markers differ between the two regimens. The aim of the present study was to explain changes in bone turnover markers using once-weekly teriparatide with a simulation model. Temporary increases in bone formation markers and subsequent decreases were observed during once-weekly teriparatide treatment for 72 weeks. These observations support the hypothesis that repeated weekly teriparatide administration stimulates bone remodeling, replacing old bone with new bone and leading to a reduction in the active remodeling surface. A simulation model was developed based on the iterative remodeling cycle that occurs on residual old bone. An increase in bone formation and a subsequent decrease were observed in the preliminary simulation. For each fitted time point, the predicted value was compared to the absolute values of the bone formation and resorption markers and lumbar BMD. The simulation model strongly matched actual changes in bone turnover markers and BMD. This simulation model indicates increased bone formation marker levels in the early stage and a subsequent decrease. It is therefore concluded that remodeling-based bone formation persisted during the entire treatment period with once-weekly teriparatide.

## Introduction

The recent development of bone anabolic drugs has provided a novel option for preventing osteoporotic fractures. The following two treatment regimens are currently approved: a once-daily 20-μg teriparatide regimen and a once-weekly 56.5-μg teriparatide regimen available in the EU, the United States and Japan. Clinical studies have demonstrated that teriparatide (human parathyroid hormone 1–34) significantly increases bone mineral density (BMD) and reduces vertebral fracture incidence with both a daily 20-μg treatment regimen (relative risk reduction: 65%)^1^ and a once-weekly 56.5-μg treatment regimen (relative risk reduction: 80%).^2^ The fracture risk reduction is partially explained by changes in BMD^3–6^ and/or bone turnover markers.^7^

Teriparatide administration is associated with a particular sequence of changes in bone turnover markers. Daily exposure to teriparatide increases bone formation markers first (e.g., procollagen type I N-terminal propeptide (P1NP)), followed thereafter by an increase in bone resorption markers (e.g. crosslinked C-telopeptide of type I collagen (CTX)).^8^ The early increase in bone formation with the use of daily teriparatide creates an ‘anabolic window’.^9,10^ In contrast, once-weekly teriparatide shows a very different pattern, with an increase in bone formation markers accompanied by a reduction in bone resorption makers.^2^ Furthermore, the increase in bone formation markers is temporary, with the peak at 4 weeks, gradually returning to and falling below baseline levels. An increase at an early stage of treatment and a subsequent decrease in bone formation markers were also observed with full length PTH 1–84 and PTH-related protein.^11,12^ Moreover, BMD continues to increase during the period when markers of bone formation are decreasing. Even though the dynamics of bone turnover markers differ between daily and weekly administration of teriparatide, increases in BMD and reductions in incident vertebral fracture are comparable. These differences in skeletal dynamics prompted us to consider theoretical models to explain the mechanism of action of once-weekly teriparatide.

Bone turnover is regulated mainly by bone remodeling, a process that involves bone-resorbing osteoclasts and bone-forming osteoblasts.^13^ The remodeling process begins with the differentiation and activation of mature osteoclasts.^14,15^ Activated osteoclasts preferentially attach to older bone surfaces.^15^ These cells excavate a unit of bone, thus creating a resorption cavity.^14,15^ New bone is formed by osteoblasts that migrate into the resorption cavity. Osteoblasts produce and deposit type I collagen and other bone matrix proteins, which are then mineralized. The remodeling unit spans a 3- to 6-month period in normal human subjects, but can be as long as 1 year or more in low turnover states, a feature of some patients with osteoporosis.^16^ Treatment with drugs for osteoporosis can also change the length of the remodeling period.^14^

Single administration of 56.5-μg teriparatide causes an immediate, transient increase in bone resorption and a decrease in bone formation, followed by increased bone formation and decreased resorption for at least 1 week.^17^ These changes describe an osteoanabolic bone remodeling cycle. Although bone resorption is eventually stimulated, bone formation is generally greater than bone resorption. Moreover, the short time changes in bone turnover markers after once-weekly teriparatide injection repeatedly showed the same direction and level of response for 24 weeks.^18^ To accommodate this observation in the setting of an osteo-anabolic effect on bone, we hypothesized that repeated teriparatide administration reduces the active remodeling surface as old bone becomes a progressively smaller component of the total bone surface.

Based on this premise, a new simulation model to help account for the osteoanabolic actions of once-weekly teriparatide is presented.

## Materials and methods

The simulation analyses were based on the results of the Teriparatide Once-Weekly Efficacy Research (TOWER) trial, which was a randomized, multicenter, double-blind, placebo-controlled trial conducted in Japan.^2^ Randomly assigned subjects (*n*=288) received once-weekly injections of teriparatide (56.5 µg) for 72 weeks. Lumbar BMD was measured using dual-energy X-ray absorptiometry at baseline and at 24, 48 and 72 weeks. For measurement of bone turnover markers, serum and urine samples were taken before teriparatide administration at baseline, at 1 week after administration, and at 4, 12, 24, 48 and 72 weeks under non-fasting conditions. The measurements of the bone formation marker (serum; s-P1NP) and the bone resorption marker (urinary crosslinked *N*-telopeptide of type I collagen; u-NTX) were performed centrally in a single batch at a validated institution (Mitsubishi Chemical Medience, Tokyo, Japan).^2^ The inter-assay CVs were 2.7%–5.0% and 6.9%–11.1%, respectively. CTX was not measured in this study.

**Figure 1:**
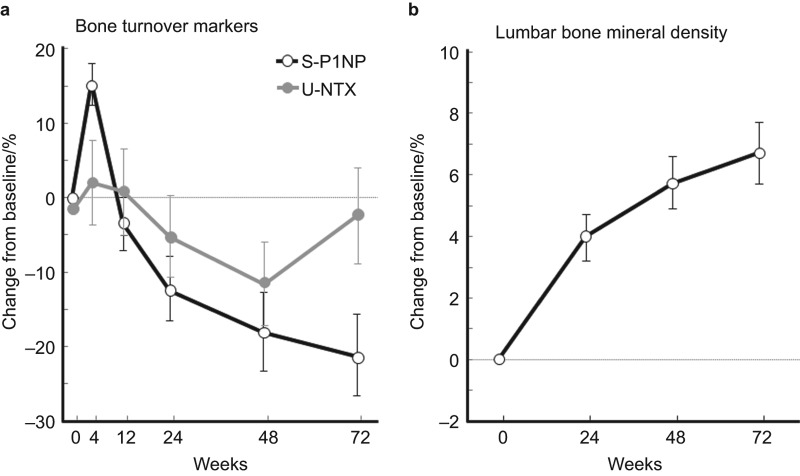
Percent change in bone turnover markers and bone mineral density in the TOWER trial (mean±95% CI). S-P1NP, serum procollagen type I N-terminal propeptide; U-NTX, urinary crosslinked *N*-telopeptide of type I collagen.

Time-dependent changes in bone turnover markers (s-P1NP and u-NTX) and lumbar BMD are shown in Figure 1.

### Preliminary simulation

**Figure 2:**
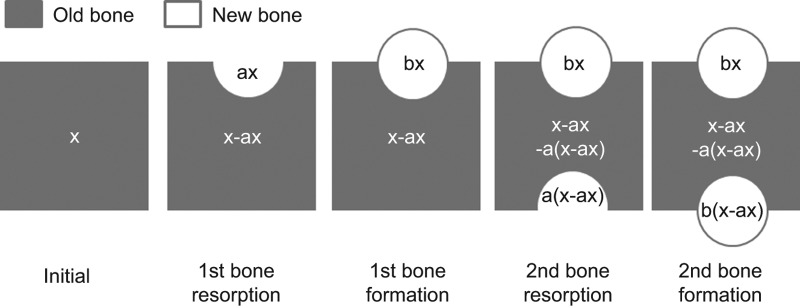
Sketch of the bone cycle. First, bone resorption occurs, and subsequently bone formation occurs on old bone. In the next step, the bone cycle occurs on the remaining old bone.

A schematic diagram illustrating the first simulation model is shown in Figure 2.

The simulation considers the cyclic formation process of mineralized absolute bone amount *x*. The model has two parameters, *a* and *b*, defined as follows:

*a*: bone resorption rate (0<*a*<1)

*b*: bone formation rate (0<*b*<1).

Prerequisite conditions were based on the following:

- Bone remodeling is a feature of mature bone.
- Bone formation occurs following bone resorption (coupling), resulting in new bone.
- With teriparatide treatment, the bone resorption rate constant *a* is smaller than the bone formation rate constant *b* (0<*a*<*b*).
- After the first remodeling cycle is completed, the subsequent cycle occurs on the residual old bone. The process is iterative, with each successive remodeling cycle focused on remaining mature bone.

Formulation (*x*: initial absolute amount of old bone, *t*: cycle number of bone remodeling)

[First cycle]

(1a)Bone resorption volume: R1=ax

(1b)Bone formation volume: F1=bx

(1c)Residual old bone volume: X1=x−R1=(1−a)x

[Second cycle]

(2a)Bone resorption volume: R2=aX1=a(1−a)x

(2b)Bone formation volume: F2=bX1=b(1−a)x

(2c)Residual old bone volume: X2=x−R1−R2=(1−a)2x

[*t*th cycle]

(3a)Bone resorption volume: Rt=a(1−a)t−1x

(3b)Bone formation volume: Ft=b(1−a)t−1x

(3c)Residual old bone volume: Xt=(1−a)t

### Plenary simulation

For a more detailed simulation, the rate parameters *a* and *b* for resorption and formation are denoted by *r*_R_ and *r*_F_, and the following three parameters were added to the simulation described above.

*f*_MAT_: fraction of new bone volume that converts to old bone (maturation) within 1 cycle (0<*f*_MAT_<1).

*f*_BMD_: fraction of new bone volume that contributes to measurement of BMD (0<*f*_BMD_<1).

*k*: the proportion of old bone volume at the initial stage (0<*k*<1).

With these parameters (*r*_R_, *r*_F_, *f*_MAT_, *f*_BMD_, *k*) given, sequences of old bone volume *V*_OLD_(*t*), as well as of new bone volume *V*_NEW_(*t*), were simulated for discrete time points *t* (*t=*0, 1, 2, 3, …, 18) according to the following formulae:

(4)VOLD(t+1)=(1−rR)VOLD(t)+fMAT VNEW(t)

(5)VNEW(t+1)=rFVOLD(t) + (1−fMAT) VNEW(t)

by which the BMD value is determined as the representative bone volume by

(6)BMD(t)=VOLD(t) + fBMD VNEW(t)

and the bone markers for formation and resorption are determined as

(7)P1NP(t+1)=rFVOLD(t)

(8)NTX(t+1)=rRVOLD(t)

Numerical values were assigned for each parameter in the following ranges: *r*_R_: 4%–31% (by 3%), *r*_F_: 8%–32% (by 3%), *f*_MAT_: 1%–10% (by 1%), *f*_BMD_: 10%–80% (by 10%) and *k*: 85%–95% (by 5%). The *r*_R_ and *r*_F_ values were based on the percent changes of bone formation (20%) and resorption (−20%) markers in the TOWER trial.^2^ *f*_MAT_ was based on the change of mineralized surface/bone surface (3.4%) in the histomorphometric data from once-weekly teriparatide treatment, and *k* was based on the baseline value of osteoid surface/bone surface (12.1%).^19^ The ranges were assigned to include these values. Since an appropriate reference value of *f*_BMD_ was not available, a wide range was assigned (10%–80%).

The predicted values were compared with the actual absolute values of P1NP, NTX and lumbar BMD for each fitted time point simulated according to the above formula with 12 960 combinations of the five parameters. To evaluate the differences between the simulated values and the actual measured values, the likelihood method based on the correlated error model assuming cluster determined by subject unit was used. The likelihood model is explained in the appendix (Supplemental file). The results of the likelihood difference across combinations of each parameter are shown in Figure 1S. Likelihood is not affected by the initial rate of old bone (*k*). Lower likelihood differences (strong matches) were observed with 1 % - 4% for *f*_MAT_ and 80% - 90% for *f*_BMD_.

## Results

### Preliminary simulation

**Figure 3:**
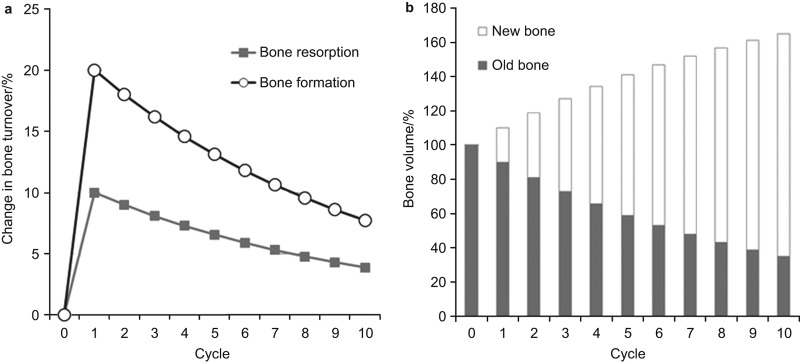
Preliminary simulation curves of bone formation, resorption and bone volume. (**a**) Changes of bone formation and resorption; (**b**) change of bone volume.

Simulated changes in formation and resorption over the course of the turnover cycles are shown in Figure 3. The values of the parameters used for the simulation were: old bone=100, resorption rate=10% and formation rate=20%. The higher bone formation rate observed early in the course of therapy falls as the turnover cycles proceed. The change in bone volume does not completely match the change in BMD. BMD is evaluated by absorptiometry with the value corresponding to the relative degree of mineralization.^18^ Although the mass of older, mature bone contributes to BMD, newly produced bone might also affect BMD. Moreover, over time, newly produced bone matures and contributes more importantly to the measurement of bone mass. Therefore, a simulation model that took into account the time-dependent contribution of newly formed, but maturing bone to BMD was generated next.

### Plenary simulation

**Figure 4:**
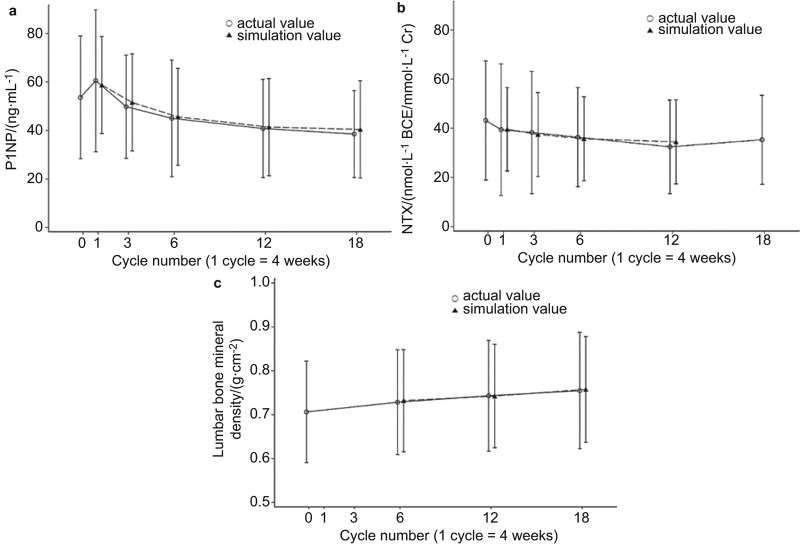
Comparison of the simulation and actual changes of bone turnover markers and bone mineral density. (**a**) Bone formation marker (P1NP); (**b**) bone resorption marker (NTX); (**c**) lumbar bone mineral density.

Figure 4 shows the simulation curve and the actual absolute changes of bone turnover markers and BMD using each parameter: *r*_R_=19%, *r*_F_=23%, *f*_MAT_=2%, *f*_BMD_=80% and *k*=85%. These simulation models match with high fidelity the changes in directly measured values.

## Discussion

Clinical trial data demonstrate that once-weekly administration of teriparatide increases bone formation markers for a short 4-week period followed by reductions to and below baseline values.^2^ Unlike daily administration of teriparatide, bone resorption markers do not increase but trend below baseline values.^8^ The gap in time between the larger and earlier increase in bone formation markers and the delayed increase in bone resorption markers observed with the administration of daily teriparatide is considered to induce an ‘anabolic window’.^10^ Although the ‘anabolic window’ is a reasonable explanation of the anabolic effect of daily teriparatide, it does not explain the anabolic actions of weekly teriparatide. To explain the anabolic actions of once-weekly teriparatide, novel simulation models were generated.

The present model strongly matches the actual changes in bone turnover markers and BMD with once-weekly teriparatide treatment. In particular, the increase in the early stages and subsequent decreases in bone formation are consistent with the model. Since the peak of P1NP was observed after 4 weeks of treatment, the simulation model assumes a remodeling cycle of 4 weeks. The peak of P1NP was also observed at 1 month with the daily teriparatide treatment regimen.^8^ Therefore, this simulation model may also help to explain this element of daily teriparatide treatment. Increases and subsequent decreases in bone formation markers were also observed in PTH 1–84 and PTH-related protein;^11,12^ interestingly, it was observed that anti-sclerostin antibody (romosozumab) increases and subsequently decreases bone formation markers such as P1NP.^20^ Nevertheless, lumbar spine BMD continues increasing throughout the treatment period. The relationship between the changes in bone formation markers and BMD is similar between once-weekly teriparatide and with anti-sclerostin antibody.

The short turnover cycle in this simulation model is not consistent with the classical bone remodeling period. Eriksen *et al.*^21^ reported that completed remodeling periods were 330 days in patients with osteoporosis and exceeded 1 000 days in patients with osteoporosis treated with fluoride, calcium and vitamin D_2_. He also divided the remodeling period into five phases: osteoclastic resorption (8 days in the normal population), mononuclear resorption phase (34 days), pre-osteoblast-like cell phase (9 days), initial mineralization lag time (15 days) and mineralization phase (130 days).^22^ The periods representing the preosteoblast-like cell phase and initial mineralization lag time agree with the time to reach the peak bone formation marker level after the start of weekly teriparatide. It is likely, therefore, that the cycle in this simulation model indicates the preosteoblast-like cell phase plus the initial mineralization lag time.

This simulation model was developed based on the hypothesis that teriparatide treatment stimulates bone remodeling on older, mature bone, and that the area of old bone is gradually reduced as new bone becomes a greater portion of bone surface. Remodeling on older mineralized bone is a mechanism by which calcium homeostasis, including microdamage repair, is maintained.^23^ The former is called non-targeted (stochastic) remodeling, and the latter is called targeted remodeling. About 30% of bone remodeling is believed to be targeted.^23^ It is well known that PTH regulates calcium balance by stimulating osteoclasts, as in stochastic remodeling. However, targeted remodeling, through the microdamage detection network of the osteocyte,^24,25^ is influenced by intermittent administration of PTH.^26^ It seems reasonable to conclude, therefore, that intermittent teriparatide administration accelerates both stochastic and targeted remodeling.

These simulation models do have limitations. First, it was not possible to fully simulate teriparatide-induced dynamics of bone resorption markers in the later stage of therapy. Once-weekly teriparatide treatment increased NTX from 48 to 72 weeks. This increase was not reproduced in the simulation model. Gatti *et al.*^27^ reported that long-term treatment with teriparatide increased the expression of Wnt antagonists such as Dickkopf-1 at 12 months after initiation of treatment. Moreover, RANKL produced by osteocytes under teriparatide stimulation may increase osteoclast formation.^28^ The mechanism of this later bone resorption marker increase may be explained by changes in the amounts or ratios of Dickkopf-1 and RANKL.

A second point is that the simulation models do not take into account compartment-specific effects of teriparatide in terms of cortical and trabecular bone. While the rate of bone remodeling is greater on trabecular surfaces, most bone is cortical. Short-term therapy, therefore, is more likely to represent the trabecular effects of teriparatide.

A third point is that serum CTX was not measured in the original TOWER trial. It was reported that the coefficient of variation of serum CTX was much smaller than that of urinary NTX. However, we previously reported that the profiles of the 24-h changes in urinary NTX with once-weekly teriparatide were almost the same in each collection week for 24 weeks.^18^ Therefore, urinary NTX may explain the change of bone resorption. Finally, this simulation model did not result in much change in bone formation markers with daily teriparatide. Further analysis and a simulation model are needed to explain this.

## Conclusion

A new model that simulates and accounts for the changes in bone turnover markers and BMD in the context of once-weekly teriparatide treatment was presented.

## Notes

Supplemental file

Figure 1S
