# Supplementary material for: New simulation model for bone formation markers in osteoporosis patients treated with once-weekly teriparatide
Source: Bone Res. 2014 Dec 23;2:14043. doi: 10.1038/boneres.2014.43 (PMC4472137; doi:10.1038/boneres.2014.43)
Supplement: Supplemental file [file boneres201443-s1.pdf]

Appendix

Likelihood model for evaluating simulated values with original TOWER trial data.

Observed variables in the TOWER trial are modeled separately by BMD, P1NP, and NTX.

For each subject, indexed by *i*, the model is given by the following formulae:

$$\mathrm{BMD}_i(t) = \alpha_1 + \beta_1 z(t) + S_{1i} + e_{1i}(t),$$

$$\text{where} \quad S_{1i} \sim \mathbf{N}[0, \sigma_1] \quad \text{and} \quad e_{1i}(t) \sim \mathbf{N}[0, \tau_1], \quad t = 6, 12, 18. \qquad \text{(A1)}$$

$$\mathrm{P1NP}_i(t) = \alpha_2 + \beta_2 (\mathbf{b}{*}x(t-1)) + S_{2i} + e_{2i}(t),$$

$$\text{where} \quad S_{2i} \sim \mathbf{N}[0, \sigma_2] \quad \text{and} \quad e_{2i}(t) \sim \mathbf{N}[0, \tau_2], \quad t = 1, 3, 6, 12, 18. \qquad \text{(A2)}$$

$$\mathrm{NTX}_i(t) = \alpha_3 + \beta_3 (\mathbf{a}{*}x(t)) + S_{3i} + e_{3i}(t),$$

$$\text{where} \quad S_{3i} \sim \mathbf{N}[0, \sigma_3] \quad \text{and} \quad e_{3i}(t) \sim \mathbf{N}[0, \tau_3], \quad t = 1, 3, 6, 12. \qquad \text{(A3)}$$

The error terms $e_{hi}(t)$ are sampled from a random variable with the covariance structure being assumed as:

$$\mathbf{Cov}[e_{hi}(t), e_{h'i'}(t')] = \gamma_h, \quad (> 0) \quad \text{when} \quad h = h' \quad \text{and} \quad i = i', \quad (t \neq t')$$

$$= 0 \qquad \text{otherwise,}$$

$$\text{for } h, h' = 1,2,3,\ t = 1, 3, 6, 12, 18, \quad \text{and} \quad i = 1, 2, \ldots, n. \qquad \text{(A4)}$$

Here $\alpha_{h,}$ and β_h are the intercept and slope for fitting linear models for each. The subjects factor $\{S_{hi}\}$ is assumed to have random effects with mean zero, standard deviation σ_h. The standard deviation of error terms is τ_h. Error terms are assumed to be independent among the three models. The error terms have common covariance values γ_h over distinct times in each model.

The likelihood function is written in the form of

$$L[\boldsymbol{\theta} \mid \{\mathrm{BMD}_i(t), \mathrm{P1NP}_i(t), \mathrm{NTX}_i(t)\}_{i = 1, \ldots, n}]$$

$$= L_1[\theta_1, r_\mathrm{R}, r_\mathrm{F}, f_\mathrm{MAT}, f_\mathrm{BMD}, k \mid \{\mathrm{BMD}_i(t)\}] * L_2[\theta_{2,}, r_\mathrm{R}, r_\mathrm{F}, f_\mathrm{MAT}, f_\mathrm{BMD}, k$$

$|\{\mathrm{P1NP}_i(t)\}]$

$$* L_3[\theta_3, r_R, r_F, f_{MAT}, f_{BMD}, k \,|\{\mathrm{NTX}_i(t)\}], \quad \text{(A5)}$$

where $\boldsymbol{\theta}$ indicates a vector of all parameters to be estimated, namely

$$\boldsymbol{\theta} = (r_R, r_F, f_{MAT}, f_{BMD}, k, \theta_1, \theta_2, \theta_3), \text{ where } \theta_h = [\alpha_h, \beta_h, \sigma_h, \tau_h] \quad \text{for} \quad h = 1, 2, 3. \quad \text{(A6)}$$

L_1, L_2, and L_3 are the normal distribution likelihoods for corresponding measurement values. The value of L is compared for different combinations of (r_R, r_F, f_{MAT}, f_{BMD}, k) to determine the best combination.
