# Supplementary figures and images for: New simulation model for bone formation markers in osteoporosis patients treated with once-weekly teriparatide
Source: Bone Res. 2014 Dec 23;2:14043. doi: 10.1038/boneres.2014.43 (PMC4472137; doi:10.1038/boneres.2014.43)

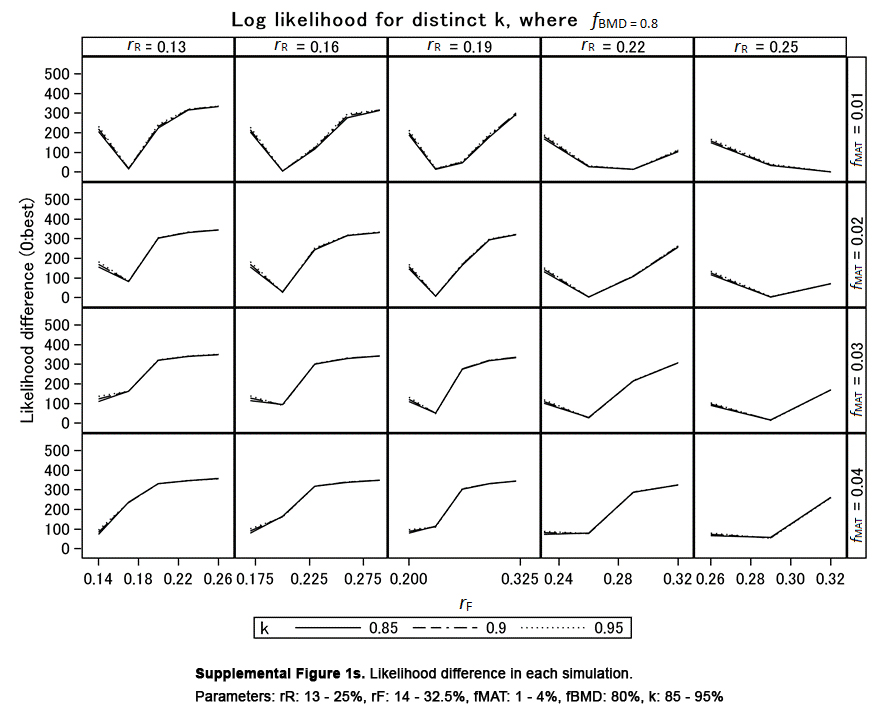

Supplement: Figure 1S [file boneres201443-s2.jpg]
